# Valence Effects on Episodic Memory in Young and Old Adults Following Exposure to Emotional Stimuli

**DOI:** 10.1111/ejn.70041

**Published:** 2025-03-03

**Authors:** Marianna Constantinou, Ala Yankouskaya, Hana Burianová

**Affiliations:** ^1^ Department of Psychology Bournemouth University Poole UK; ^2^ School of Psychology Swansea University Swansea UK

**Keywords:** ageing, arousal, fMRI, PLS, retrieval

## Abstract

Episodic memory benefits from arousal, with better retrieval linked to arousing to‐be‐remembered information. Arousal's impact on subsequent memory processes, particularly for nonarousing stimuli, remains unclear. Healthy ageing is associated with emotion regulation changes and declines in episodic memory, which may influence how arousal affects memory processes. This functional Magnetic Resonance Imaging (fMRI) study examined the effects of valence on episodic memory in young and old adults, focusing on memory of neutral information following arousal exposure. Neural activity was assessed at three time points: during exposure to arousing and nonarousing images, encoding of neutral videos following image exposure, retrieval of the encoded videos. We hypothesised that valence would induce distinct neural activation across task stages, and exposure to negative stimuli would be associated with worse retrieval. Old adults were expected to show stronger neural responses to positive valence and less disruption from negative valence on memory performance. Behavioural results revealed that only negative valence was associated with impaired retrieval. fMRI results replicated age‐related differences in memory performance, with old adults compensating through increased hippocampal and frontal gyri activity. Negative valence was associated with increased activity in the occipital cortex and precentral gyri, also affecting upcoming encoding with heightened activity in the left insula, precuneus and middle temporal gyrus. In old adults, positive valence prompted increasing neural engagement from initial exposure to retrieval, reflecting changes in emotion regulation strategies. Findings emphasise the enduring impact of negative valence on subsequent cognitive processes and suggest that age‐related changes in emotional regulation influence memory‐related neural processes.

## Graphical abstract

Young and old adults exposed to positive, negative or neutral stimuli showed worse episodic memory following negative valence. Negative stimuli increased neural activity during exposure with lasting effects on subsequent encoding. In old adults, while they failed to neurally differentiate between positive and negative stimuli, positive valence increased its neural impact from initial valence exposure to unrelated retrieval, suggesting a dynamic shift influenced by emotion regulation changes in ageing.

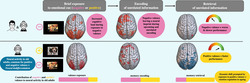

**Abbreviations**
BOLD: blood oxygenation level‐dependent
CRUNCH: compensation‐related utilisation of neural circuits hypothesis
eCMR: emotional context maintenance and retrieval model
fMRI: functional magnetic resonance imaging
GAPED: Geneva Affective Picture Database
LRT: likelihood ratio test
LV: latent variable
MMSE: Mini‐Mental State Examination
MNI: Montreal Neurological Institute
OASIS: Open Affective Standardized Image Set
PLS: partial least squares
RTs: response times
TR: repetition time

## 1 Introduction

Episodic memory, encompassing the retrieval of experiences rich in contextual details (Tulving 1972), is influenced by emotionally salient stimuli (Dolcos et al. 2017). Salience refers to the distinctiveness of a stimulus from its surroundings (Uddin 2015) and affects episodic memory through arousal. Arousal denotes the intensity of the stimulus's perceived impact (i.e., high or low; Alger et al. 2018) and is typically defined in relation to its valence (i.e., positive, neutral and negative; Alger et al. 2018). Episodic memory of to‐be‐remembered high‐arousing, compared to low‐arousing stimuli, is associated with better performance, that is, higher accuracy and faster response times during memory retrieval (Constantinou et al. 2023; Kensinger et al. 2007; Liu et al. 2008; Ritchey et al. 2011; Schümann et al. 2018; Sharot and Phelps 2004). Moreover, high‐arousing information enhances the subjective sense of recollection (Phelps and Sharot 2008), presumably driven by increased and prolonged fixations it attracts (Astudillo et al. 2018; Bradley et al. 2011). According to the emotional context maintenance and retrieval model (eCMR; Talmi et al. 2019) enhanced attention to emotional items reinforces connections between these items and their source context, improving information binding. Importantly, this heightened attention is not restricted to the arousing stimulus alone but may also extend to the retrieval of adjacent neutral information both before and after the presentation of arousing stimuli (Clewett and McClay 2024; Lee et al. 2015; Sakaki et al. 2014). Specifically, negative stimuli disrupt the memory for the order of items in a sequence but enhance memory for items within the same context (Clewett and McClay 2024). Negative valence further weakens associative binding, reducing memory coherence for nearby neutral objects (Bisby et al. 2016). This suggests that negative valence enhances memory within a shared negative context, while impairing memory for adjacent neutral items.

The effect of arousing stimuli is also observed at the neural level, with heightened whole‐brain activity during retrieval, compared to stimuli of lower arousal. Increased responses are found in the anterior temporal pole, amygdala, hippocampus and entorhinal cortex, which persist even after a 1‐year retention period (Dolcos et al. 2004, 2005). A neural differentiation driven by valence is indicated by enhanced activity in the prefrontal and orbitofrontal regions bilaterally, and the left anterior temporal pole for positive contexts, and enhanced activity in the right dorsolateral prefrontal cortex, posterior cingulate cortex, left hippocampus and amygdala for negative contexts (Maratos et al. 2001). Interestingly, negative stimuli, compared to neutral stimuli, are associated with increased amygdala activity but diminished hippocampal involvement (Bisby et al. 2016). This does align with the notion that the amygdala primarily participates in item‐emotion bindings, which contributes to the slower decay of emotional items, but the hippocampus is more involved in item‐context bindings (Yonelinas and Ritchey 2015). Such evidence could explain attentional narrowing caused by negative items, involving central/peripheral trade‐offs, with heightened focus on central aspects and reduced attention to peripheral details of a stimulus (Chipchase and Chapman 2013). Therefore, arousing stimuli are associated with increased neural activity during retrieval, which further differentiates based on emotional valence, reinforcing the need to examine the individual contributions of negative and positive triggers.

Healthy ageing is associated with changes in both emotion‐related and memory‐related processes, which may affect how arousing stimuli are perceived and subsequently remembered. Old adults often exhibit differences in emotion regulation processes, with some studies suggesting a positivity bias in healthy ageing (Mammarella et al. 2016; Mather and Knight 2005). This shift in emotional processing makes it critical to investigate how valence can influence memory processes in old adults, as it can potentially enhance memory performance through binding mechanisms. Previous research remains limited, with some studies indicating that the impact of arousal on memory may diminish with age (Lee et al. 2023) or even disappear (Nashiro and Mather 2011). Old adults compared to young adults exhibit distinct neural patterns when processing emotional stimuli. Specifically, when viewing positive versus neutral images, old adults show increased activity in the left amygdala, middle temporal and right lingual gyrus, alongside decreased activity in bilateral occipital cortex, inferior parietal lobule and supplementary motor area (Kehoe et al. 2013). This is also accompanied by old adults rating images as more positive than young adults (Kehoe et al. 2013). Similarly, old adults report lower emotional intensity for negative images despite showing right amygdala activity in response to high‐arousing stimuli, as in young adults (Dolcos et al. 2014). These age‐related differences in emotional processing emphasise the need to examine how emotional valence influences memory in old adults, particularly as they experience declines in episodic memory (Kinugawa et al. 2013; Korkki et al. 2020; St‐Laurent et al. 2011). Age‐related changes in emotion regulation suggest that while negative stimuli might still elicit an influence on memory processes due to their inherent emotional impact, old adults' tendency to regulate their emotions might modulate how these effects are experienced. Conversely, positive stimuli might become more salient in ageing due to the greater focus engaged by old adults on positive events. Arousing stimuli, both positive and negative, engage different neural networks, involved in attention (dorsal attention network), emotional relevance (salience network) and memory retrieval (default mode network), across a broad age range (18–88 years, mean age = 52 years; Katsumi and Moore 2022), suggesting that valence influences how memory is processed and retrieved across the lifespan. However, given that old adults often regulate their emotions by prioritising positive information (Isaacowitz et al. 2006), this could lead to unique interactions prompted by age. Moreover, since arousal is associated with better episodic retrieval in young adults, understanding its role in old adults could provide insights into potential mechanisms for counteracting memory decline in healthy ageing.

In the current study, we aimed to investigate the influence of valence on the retrieval of subsequent unrelated information, focusing specifically on content devoid of emotional elements. A three‐stage task was used, which exposed participants to positive, negative or neutral image cues, followed by nonarousing videos to allow episodic memory encoding and concluded with an assessment of memory retrieval through statements, which related to the video content. The objectives were to examine the distinct effects of negative and positive valence on episodic memory and to investigate performance differences between young and old adults. These objectives were assessed behaviourally, measuring accuracy and response times, and neurally, examining whole‐brain activity across the different task stages. We tested three hypotheses: (i) positive and negative valence will elicit distinct neural activity patterns during each task stage (image presentation, video encoding and memory retrieval). These neural effects were assessed using task partial least squares (PLS; Mcintosh et al. 1996) analysis, which examined whole‐brain activity patterns separately for each stage to capture dynamic valence‐driven changes over time (from initial valence exposure, to encoding of unrelated details, to retrieval of those details); (ii) positive and negative valence will differentially influence behavioural performance, with worse memory retrieval being associated with prior exposure to negative valence; (iii) age‐related differences will impact the influence of positive and negative valence, with old adults showing a stronger neural response to positive valence across task stages (consistent with the positivity effect in ageing; Isaacowitz et al. 2006). Moreover, negative valence is expected to have a weaker disruptive effect in old adults' behavioural performance given their tendency towards emotion regulation processes and the prioritisation of positive information. Neurally, these effects were assessed using task PLS, comparing patterns between age groups and within each group separately. By testing these hypotheses, we intend to examine how negative and positive stimuli influence memory processes from initial exposure to encoding and retrieval, comparing their effects in young and old adults.

## 2 Methods

### 2.1 Participants

The final sample size after data preprocessing comprised 26 young adults (mean age = 24 years; SD = 3.6; 8 males) and 20 old adults (mean age = 69 years; SD = 5.4; 9 males). Thirty‐three young adults and 21 old adults were recruited. Inclusion criteria ensured normal or corrected‐to‐normal vision, no colour vision deficiencies, the absence of neuropsychiatric disorders or head trauma and a history without medications affecting cardiovascular responses (e.g., antidepressants). All participants had a minimum education level of a higher education diploma. Old adult participants demonstrated cognitive intactness, as indicated on the Mini‐Mental State Examination (MMSE; Folstein et al. 1975). For MRI compatibility, participants were excluded if they had any metal implants in their body. Before conducting the study, approval was obtained from the Ethics Committee of the Psychology Department at Bournemouth University, and written consent was obtained from each subject.

### 2.2 Task Stimuli

#### 2.2.1 Image

Forty‐eight colour photographs displaying animals, humans and scenes were used as visual cues for the experimental conditions. Of these, 24 images were selected from the Open Affective Standardized Image Set (OASIS) database (Kurdi et al. 2017) to represent positive valence and 24 images from Geneva Affective Picture Database (GAPED; Dan‐Glauser and Scherer 2011) to represent negative valence.

These two distinct databases were employed to select negative and positive images due to specific content considerations and the aim for balanced emotional valence representation. The GAPED has a limited number of positive images compared to negative ones, and they also have a limited diversity of positive stimuli, making it difficult to pair and balance content across valences. On the other hand, the OASIS database offers a diverse range of positive images. To overcome limitations in comparing the images from the two databases, we chose the highest rated positive images from OASIS database (valence range 5.68–6.49; mean valence = 6.11; SD valence = 0.2) and the higher rated negative images from the GAPED (valence range 0.72–45.70; mean valence = 11.35; SD valence = 12.37). Given the GAPED ratings are on a 0–100 scale and OASIS ratings use a 7‐point Likert scale, we normalised both sets of ratings to a common scale and compared them using two independent sample *t*‐tests, which revealed significant differences between the ratings in valence (*t*(23) = 3.83, *p* < 0.001), and the lack of significant differences in arousal (*t*(23) = −1.59, *p* = 0.125).

A black‐and‐white scrambled image was used to serve as the neutral condition, lacking cueing valence effects, further eliminating any confusion and potential misinterpretation linked with neutral images of objects, faces or scenes. The stimuli were presented using PsychoPy (v2021.2.3; Peirce et al. 2019).

#### 2.2.2 Video‐Target

Twenty‐four videos were chosen from the ActivityNet database (Heilbron et al. 2015) and cropped to a duration of 7 s, striving for a balance between providing sufficient information for participants to respond to subsequent questions and minimising the potential drawbacks of extended video duration. ActivityNet provides a wide range of videos displaying common human activities. All videos depicted simple, everyday, neutral activities rather than arousing content. These videos specifically showcased individuals actively participating in routine activities, such as playing sports, music, cleaning or cooking. The pairing of the videos with the previously shown image was controlled to guarantee one video appearance per each condition to allow consistent comparisons between positive, negative and neutral conditions.

### 2.3 Experimental Design

A 2 × 3 mixed‐subjects design was used with Group as the between‐subjects factor with two levels: young adults and old adults, and Condition as the within‐subjects factor with three levels: negative valence, positive valence and neutral valence. The dependent variables included changes in the Blood oxygenation level‐dependent (BOLD) response, response times (RTs) and accuracy.

### 2.4 Task and Experimental Procedure

After providing participants with details about the study and obtaining informed consent, old adults underwent a cognitive prescreening assessment using the MMSE. None of the old adults displayed any cognitive impairment based on their performance (score range 28–30; mean score = 29.9; SD = 0.48). Following this, all participants engaged in a practice trial of the task outside the MRI scanner using a laptop.

**FIGURE 1:**
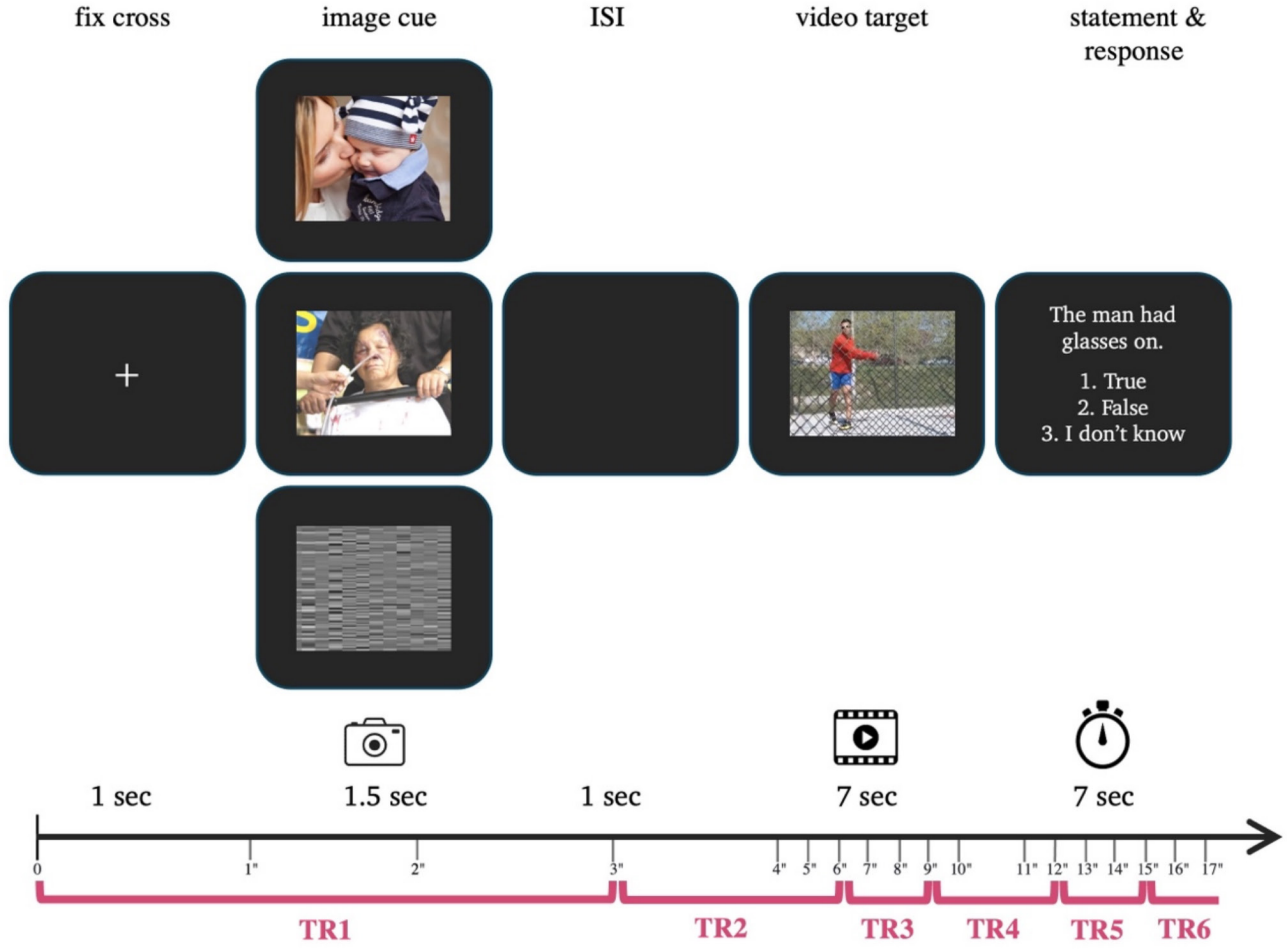
Schematic representation of the Episodic Memory (EM) task: Each trial started with the presentation of a fixation cross for 1 s, followed by an image (cue) for 1.5 s. Images were either of (i) positive valence, (ii) negative valence or (iii) neutral valence. An inter‐stimulus‐interval (ISI) followed for 1 s, and then a video (target) appeared on the screen for 7 s. Each video was presented three times in a randomised order. A statement followed, which was accompanied by three fixed possible answers: (1) True, (2) False and (3) I do not know. Participants had 7 s to respond by pressing one of the three buttons. The statement remained on the screen for the full remaining 7 s before transitioning to the next trial. The red line outlines each TR aligning with the PLS analyses for each task stage.

Upon entering the scanner, detailed instructions were given on how to position their fingers on the MRI‐compatible response box, using their index finger for ‘True’, middle finger for ‘False’ and ring finger for ‘I don't know’ responses during the task. The scanning procedure commenced with a 7‐min structural scan. Subsequently, participants were presented with the task, which consisted of 72 trials (24 per condition) spread across three functional runs. A presentation of an image was followed by a video and then a statement. Participants were allotted 7 s to respond, with only the first response being considered. Each video was presented once, paired with a different condition in each run. Functional runs were presented in a randomised order, each lasting for 7 min. When participants completed the task, they were debriefed on the study's purpose. For a schematic representation of the episodic memory task, see Figure 1.

### 2.5 Functional Magnetic Resonance Imaging (fMRI) Data Acquisition

Brain images were collected with a 3 T Siemens MAGNETOM Lumina scanner. A T1‐weighted anatomical MRI was acquired using an MP2RAGE sequence with 176 sagittal slices, TR = 4000 ms, TE = 2.98 ms, FOV = 256 mm, flip angle = 4°, and voxel size = 1 mm^3^.

BOLD contrast whole‐brain functional images were acquired using a T2‐weighted gradient‐echo Echo Planar Imaging (EPI) sequence with a 32‐ channel head coil. The acquisition parameters were as follows: TR = 3000 ms; TE = 30 ms; matrix size = 64 × 64; in‐plane resolution 2.5 × 2.5 mm and flip angle = 90 °. Four hundred twenty volumes with 46 axial slices were measured in interleaved slice order and positioned along a line to the anterior–posterior commissure (AC‐PC orientation). An automated high‐order shimming technique was used to maximise magnetic field homogeneity.

### 2.6 Behavioural Data Analysis

Outliers, defined as observations deviating more than 2.5 SDs from each participant's mean response time, were excluded, along with their corresponding accuracy responses. This approach is in line with the idea that most observations in a normal distribution cluster within a few SDs of the average, with outliers lying outside this range (Miller 1991). To account for age‐related slowing and declines in information processing speed, response times were transformed into z‐scores (Hedge et al. 2018).

### 2.7 Mixed Modelling

Mixed modelling was employed to analyse the effects of Age and Valence. This procedure offers advantages over classical analysis of variance (ANOVA) by handling missing data better and allowing estimation of fixed effects, interactions and variance and covariance components of random effects due to subjects (Baayen et al. 2008). We used a stepwise approach for model specification and comparison. Starting with a model including Age, Valence, Video repetition and relevant interactions, we systematically removed factors or interactions to find the most parsimonious model with the best fit. The final model included fixed effects Age, Valence, Video repetition and the Age × Valence interaction, along with random effects Participant and Video stimulus. The contribution of the random effect of subjects was assessed using the likelihood ratio test (LRT; Judd et al. 2012). The likelihood ratio statistic is equal to two times the difference of the log‐likelihoods of two models, where one model includes a parameter of interest (fitted model), and the second model (null‐model) does not contain the parameter of interest. In this study, the employment of mixed models proved particularly efficacious, as they adeptly manage the complexities associated with unbalanced data due to varying group sizes, ensuring accurate estimation of effects across groups (Bates et al. 2014). A generalised mixed model was used to investigate accuracy, and a linear mixed model was used for response time. Both models were estimated using RStudio (v2023.03.0; RStudio Team 2023).

### 2.8 fMRI Data Preprocessing

Four young adults and one old adult were excluded from the analyses due to software failure (*N* = 3) and incidental findings (*N* = 1). We used SPM12 (www.fil.ion.ucl.ac.uk/spm; Friston et al. 1995) in MATLAB R2022a (Mathworks, Inc. 2022) to preprocess functional neuroimaging data. The preprocessing pipeline included functional realignment (correction for head motion‐induced intervolume displacement), unwarp, slice‐timing correction (differences in acquisition time), segmentation and normalisation to the Montreal Neurological Institute (MNI) space using the default TPM MNI template and the unified normalisation–segmentation procedure. After the images were affine aligned to the MNI space, they were resliced to 2 × 2 × 2 mm and smoothed with an isotropic 6 mm FWHM Gaussian kernel. Default high‐pass temporal filtering (1/128 Hz cut‐off) in SPM12 was applied to remove low‐frequency noise and signal drifts from each voxel's fMRI time course.

### 2.9 fMRI Data Analysis

We used PLS (Mcintosh et al. 1996) analysis on the imaging data to investigate how different task demands (i.e., episodic memory retrieval at varying levels of valence) relate to changes in brain activity throughout the entire task. PLS aims to identify latent variables (LVs) that capture the maximum covariance between the fMRI data and the experimental design (Krishnan et al. 2011). The data are organised into two matrices. Matrix X represents the neuroimaging data where each row represents a subject, and each column represents a voxel in the brain. Matrix Y represents coding corresponding to the experimental design. PLS uses matrices X and Y to compute a combined a single mean‐centred matrix entered for analyses. The single matrix is decomposed using singular value decomposition, generating a set of hierarchically ordered LVs that capture common and unique patterns between brain activations and experimental design or behaviour.

Analogous to the loadings in principal component analysis, PLS uses the concept of saliences. Saliences represent the strongest relationship between a voxel and its neighbouring values obtained by decomposing the correlation matrices. For each LV, PLS provides an image of voxel silences, revealing how neural activity is influenced by experimental conditions or behaviour. It also gives a profile of task saliencies, indicative of the impact of brain activity across conditions, along with a singular value representing the percentage of covariance accounted for by the LV. All voxels and conditions are analysed together, eliminating the need for correction for multiple comparisons. PLS analyses have been used in previous studies examining neural representations during declarative memory retrieval (Burianova et al. 2010; Grady et al. 2015; St‐Laurent et al. 2011) and investigating emotion‐related processes (Dzafic et al. 2016; Keightley et al. 2003).

Here, we systematically investigated the neural dynamics of the relative differences and similarities in brain activity between conditions in young and old adults. Initially, we conducted a (i) Task‐PLS analysis that included both age groups and all conditions, aiming to explore valence effects across the two groups. However, given challenges posed by varying group sizes and potentially age‐related physiological differences, which could have impacted the results, we then proceeded to perform separate analyses for each age group. For each stage of the task—image presentation, video presentation and statement presentation—we also conducted the following analyses: (ii) Task PLS with all conditions in young adults; (iii) Task PLS with all conditions in old adults. During the image presentation stage, the goal was to identify neural activity differences associated with valence. In the video presentation stage, the focus shifted to detecting neural differences when participants viewed videos, following the initial valence exposure, aiming to understand how prior valence influenced neural activity during encoding. Finally, during the statement presentation stage, the analysis assessed whether the valence condition affected neural activity during memory retrieval. Conducting these analyses separately for each group allowed us to isolate the effects of valence on neural activity, mitigating the confounding influences of age‐related physiological changes, such as shifts in vascular function that could affect the BOLD signal (Gazzaley et al. 2005). Additionally, analysing each group separately accounted for the smaller sample size in the old group, which would prompt lower variance extraction, and potentially decrease the sensitivity and robustness of the findings, as compared to the larger young adult group.

PLS relies on precise synchronisation between the timing of experimental stimuli and the TR intervals. Specifically, each task stage (i.e., image presentation, video presentation and statement presentation) has a defined duration that aligns with specific TR intervals. For instance, the fixation cross‐ and image presentation are completed within the first TR (0–3 s), while the subsequent video presentation spans across TRs 2, 3 and part of TR 4 (3–12 s). Following this, the statement with response occurs from 10.5 to 17.5 s, encompassing parts of TRs 4, 5 and 6 (9–18 s). This alignment ensured that the neural responses corresponding to each task stage are captured distinctly within the designated TRs, enabling precise temporal analysis of brain activity associated with different experimental conditions (refer to Figure 1).

Four post hoc behavioural PLS analyses were conducted to explore the relationship between neural and behavioural performance. As in Task PLS, the accuracy and response times were examined separately for each age group, using brain–behaviour PLS analyses. The decision to conduct separate analyses aimed to disentangle neural activity associated with higher or lower performing individuals within each age group. This approach was particularly crucial, given the observed age‐related neural differences across all task stages. By conducting analyses separately for each group, we aimed to ensure that identified neural activity associated with better or worse behavioural performance was not confounded by age‐related effects, allowing a more precise examination of the relationship between neural activity and behavioural performance within each age group.

Saliences representing summary measurements of the spatial arrangement were captured for each condition across every LV. Statistical significance of each LV was assessed with permutation testing, repeated 500 times (McIntosh and Lobaugh 2004). To further evaluate the reliability of activations identified by permutation testing, bootstrapping was used and repeated 100 times (McIntosh and Lobaugh 2004). This calculated the standard error across voxel salience for each LV. Voxels with a bootstrap ratio greater than 3.0 were accounted reliable, approximating *p* < 0.001. Brain scores captured the degree of neural activity associated with the experimental conditions, reflecting how strongly activity aligned with the patterns identified by each LV. In other words, brain scores provided a numerical representation of how the brain responded to different conditions (and groups), allowing comparisons of neural activity. Confidence intervals of these brain scores for each LV were then calculated at 95%.

## 3 Results

### 3.1 Behavioural Results

#### 3.1.1 Accuracy

In the accuracy model, a fixed effect omnibus test revealed a main effect of Condition, χ^2^(2) = 13.29, *p* = 0.001. Post hoc tests with Bonferroni corrections for multiple comparisons showed that the effect was driven by significant lower accuracy scores in the Negative compared to the neutral condition (MD = 0.691, SE = 0.072, z = −3.57, *p*_bonf_ < 0.001). The mean difference between Negative and Positive conditions (MD = 0.815, SE = 0.0827, z = −2.02, *p*_bonf_ = 0.131), as well as the mean difference between Neutral and Positive conditions (MD = 1.180, SE = 0.125, z = 1.56, *p*_bonf_ = 0.353), was not significant. A main effect of Group was significant χ^2^(1) = 7.70, *p* = 0.006 and was driven by higher accuracy in the Young, compared to the Old group, SE = 0.08, z = 2.77. The Group × Condition interaction was not significant, χ^2^(2) = 2.50, *p* = 0.287.

The accuracy model further showed a main effect of Video Repetition, χ^2^(2) = 9.70, *p* = 0.008. Post hoc tests indicated significantly more accurate responses for the third compared to first video appearance, MD = 0.732, SE = 0.076, z = −3.029, *p*_bonf_ = 0.007, but not between the first and second video appearance, MD = 0.810, SE = 0.0819, z = −2.083, *p*_bonf_ = 0.112, and between the second and third video appearance, MD = 0.903, SE = 0.0951, z = −0.966, *p*_bonf_ = 1.00.

The variance of the random effect of Participant (SD = 0.373, σ^2^i = 0.139, CI [0.0678, 0.262], ICC = 0.041) and Video Stimulus (SD = 0.481, σ^2^i = 0.231, CI [0.1203, 0.470], ICC = 0.0656) was small.

**FIGURE 2:**
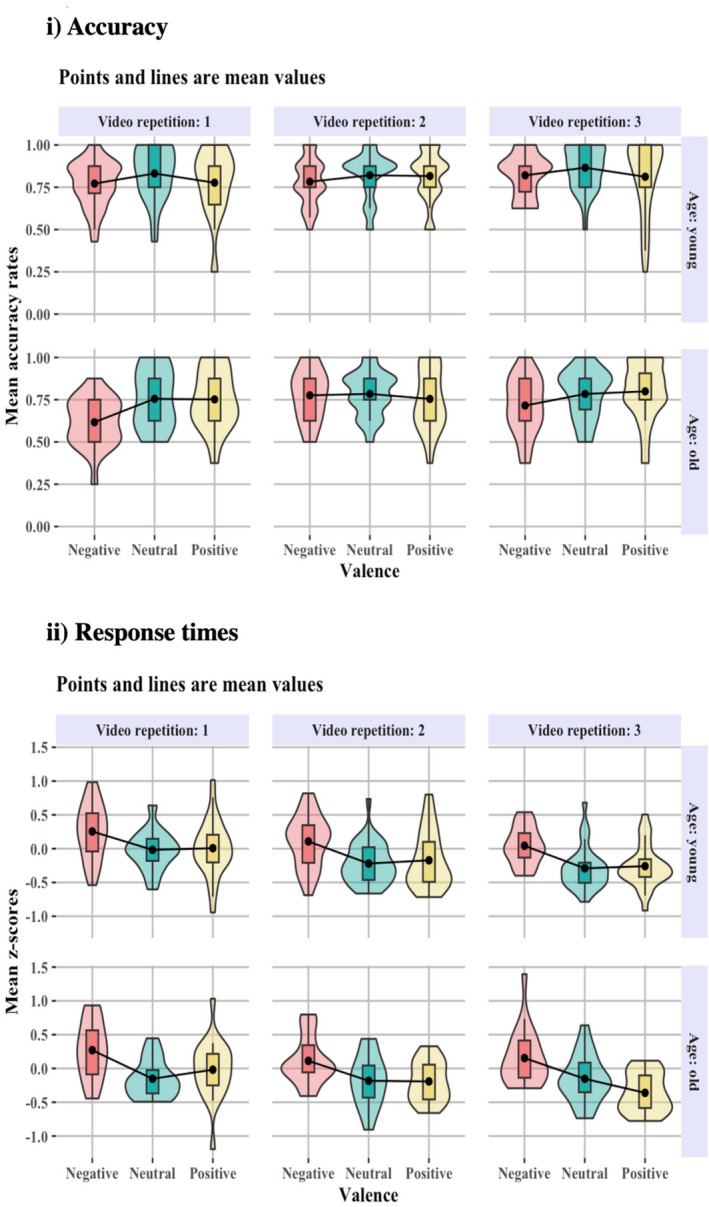
(i) Accuracy; (ii) response times of the behavioural data. Figures include accurate responses only, on outlier‐corrected data across the experimental conditions. Participants were significantly less accurate and slower for the negative condition than the neutral and positive conditions. Old adults were significantly less accurate, but not slower compared to young adults.

Overall, the behavioural evidence shows that both groups were significantly less accurate on trials that were cued with a negative, compared to neutral image, with young, compared to old, adults performing significantly better (Figure 2i).

#### 3.1.2 Response times

In the response time model, a fixed effect omnibus test revealed a main effect of Condition, F(2, 2790) = 47.05 *p* < 0.001. Post hoc tests with Bonferroni corrections for multiple comparisons indicated that the effect was driven by significant slower responses in the Negative compared to the Neutral condition, MD = 0.343, SE = 0.0402, *t*(2754) = 8.55, *p*_bonf_ < 0.001 and in the Negative compared to the Positive condition, MD = 0.337, SE = 0.0404, *t*(2760) = 8.35, *p*_bonf_ < 0.001. The mean difference between Neutral and Positive conditions was not significant, MD = −0.0058, SE = 0.0395, *t*(2763) = −0.147, *p*_bonf_ = 1.00. The main effect of Condition, F(1, 2784) = 0.0717 *p* = 0.789, as well as the Group × Condition interaction, F(2, 2784) = 0.3183, *p* = 0.727, was not significant.

The response time model further revealed a main effect of Video Repetition, F(2, 2784) = 17.9849, *p* < 0.001. Post hoc tests indicated faster response times for the second compared to first video appearance, MD = 0.158, SE = 0.039, *t*(2760) = 4.06, *p*_bonf_ < 0.001, and for the third compared to the first video appearance, MD = 0.228, SE = 0.039, *t*(2769) = 5.87, *p*_bonf_ < 0.001, but not between the second and third video appearance, MD = 0.070, SE = 0.034, *t*(2768) = 1.83, *p*_bonf_ = 0.200.

The variance of the random effect of Participant (SD = 9.42e‐9) and Video Stimulus (SD = 0.279) was small. The LRT showed that a model with the random effect of Video Stimulus explained performance significantly better compared to a model with fixed effects only (LRT = 194, df = 1, *p* < 0.001). The random effect of Participant was negligible (LRT = 5.46e‐12, df = 1, *p* = 1.00).

Overall, the behavioural evidence shows that both groups were significantly slower on trials that were cued with a negative, compared to neutral, or positive image (Figure 2ii).

### 3.2 fMRI Results

#### 3.2.1 Whole‐Brain Task‐PLS Analyses

##### 3.2.1.1 Between‐Group Differences Related to Task

In all Task‐PLS analyses (i.e., image presentation, video presentation and statement presentation), which included both young and old adults across all conditions, a consistent pattern of activity emerged. The first LVs differentiated neural activity in young adults from that in old adults, reflecting age‐related patterns in neural processing. This differentiation was found during image presentation (*p* = 0.006, accounting for 64% covariance in the data), video presentation (*p* < 0.001, accounting for 67% covariance in the data) and retrieval (*p* = 0.002, accounting for 68% covariance in the data).

In old adults, as opposed to young, during image and video presentation, we found significantly greater activity in the hippocampus bilaterally, left caudate nucleus, cuneus, supplementary motor area and regions in the right temporal gyrus. In young compared to old adults, during image encoding, we found significant heightened activity in the left middle temporal gyrus, right fusiform and left middle orbital frontal gyrus. During memory retrieval, in young compared to old adults, we report increased activity in the left caudate nucleus, right supplementary motor area, lingual gyri and cuneus, while in old compared to young adults, we found significantly increased activity in the left middle occipital gyrus.

**FIGURE 3:**
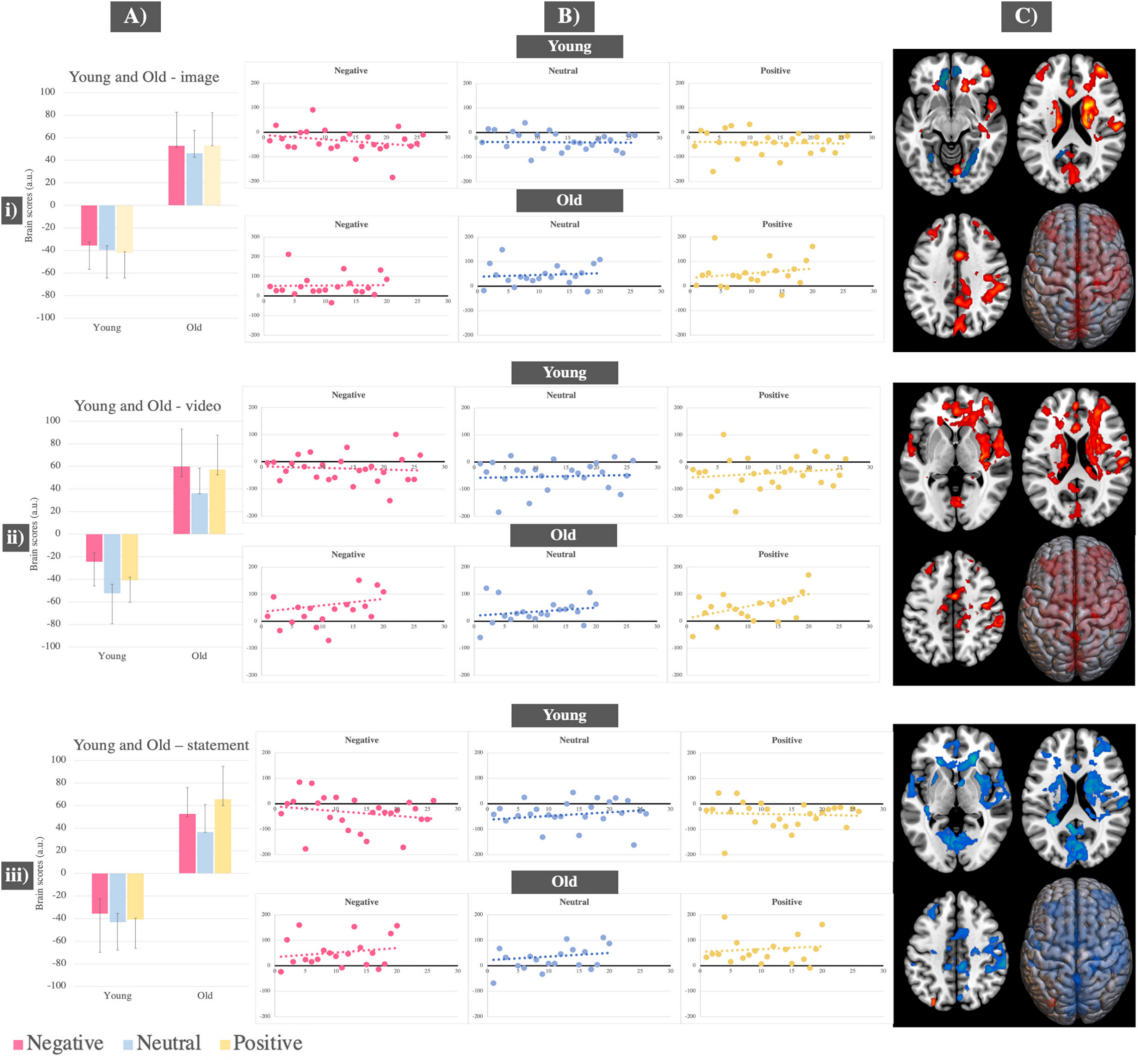
Task PLS results—First significant LVs across analyses during: (i) image presentation; (ii) video exploration; (iii) episodic memory retrieval, across all conditions in young and old adults. (A) Mean brain scores related to activity in young adults versus activity in old adults across all three conditions with error bars indicating 95% confidence intervals calculated from bootstrapping; (B) scatterplots displaying individual‐level brain scores for each condition, task stage and group, highlighting opposing patterns in neural activity between young and old adults; (C) brain figures displaying regions with significantly increased activity in old compared to young adults (represented in red) and regions with significantly increased activity in young compared to old adults (represented in blue).

Results from the first significant LV did not show significant condition differences in either group, as indicated by the overlapping confidence intervals in the bar graphs (see Figure 3 and Tables S1–S3).

##### 3.2.1.2 Between‐Group Differences Related to Valence

**FIGURE 4:**
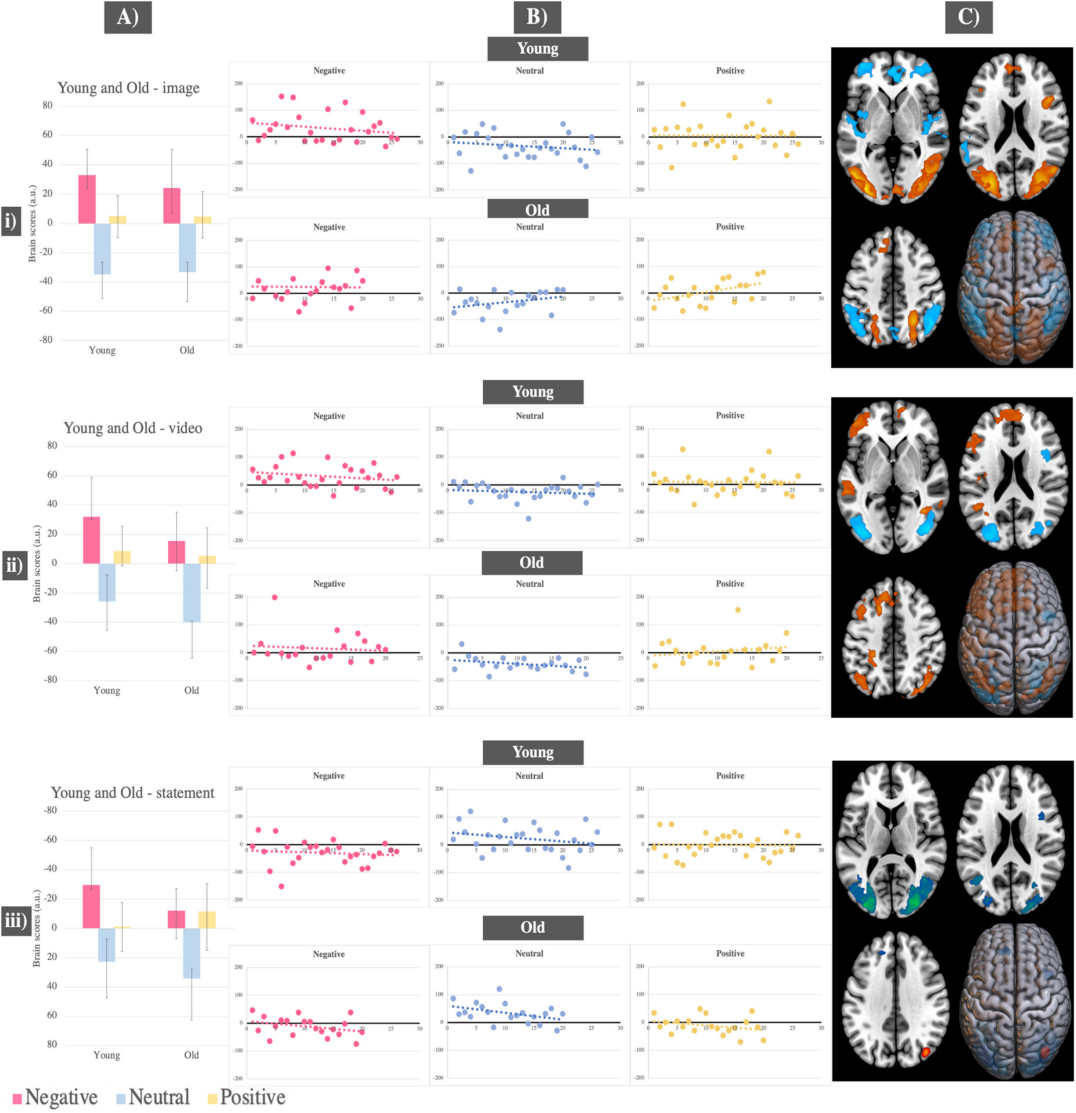
Task PLS results—Second significant LVs across analyses during: (i) image presentation; (ii) video exploration; (iii) episodic memory retrieval across all conditions in young and old adults. (A) Mean brain scores related to activity in negative versus neutral condition in young and old adults. Error bars indicating 95% confidence intervals calculated from bootstrapping; (B) scatterplots displaying individual‐level brain scores for each condition, task stage and group, highlighting differential neural patterns between the negative and neutral condition; (C) brain figures displaying regions with significantly increased activity associated with the negative condition (represented in red) and regions with significantly increased activity for the neutral condition (represented in blue) across young and old adults.

The second LVs differentiated neural activity associated with the negative condition from neural activity in the neutral condition. This differentiation was found during image presentation (*p* < 0.01, accounting for 21% covariance in the data), video presentation (*p* = 0.008, accounting for 18% covariance in the data) and retrieval (*p* = 0.006, accounting for 15% covariance in the data). The negative compared to neutral condition was associated with increased activity in the precentral gyri, regions in the inferior occipital gyri, left insula and left fusiform gyrus. The neutral condition, compared to negative condition, was linked to increased activity mostly in regions of the occipital cortex, bilaterally, during video and statement presentation, and with the right supramarginal, and superior temporal gyrus, as well as left parietal gyrus during image presentation. The second significant LV failed to reveal any significant patterns related to the positive condition, as indicated by the confidence intervals crossing 0 in the bar graphs. For results refer to Figure 4 and Tables S4–S6.

##### 3.2.1.3 Within‐Groups Analyses

**FIGURE 5:**
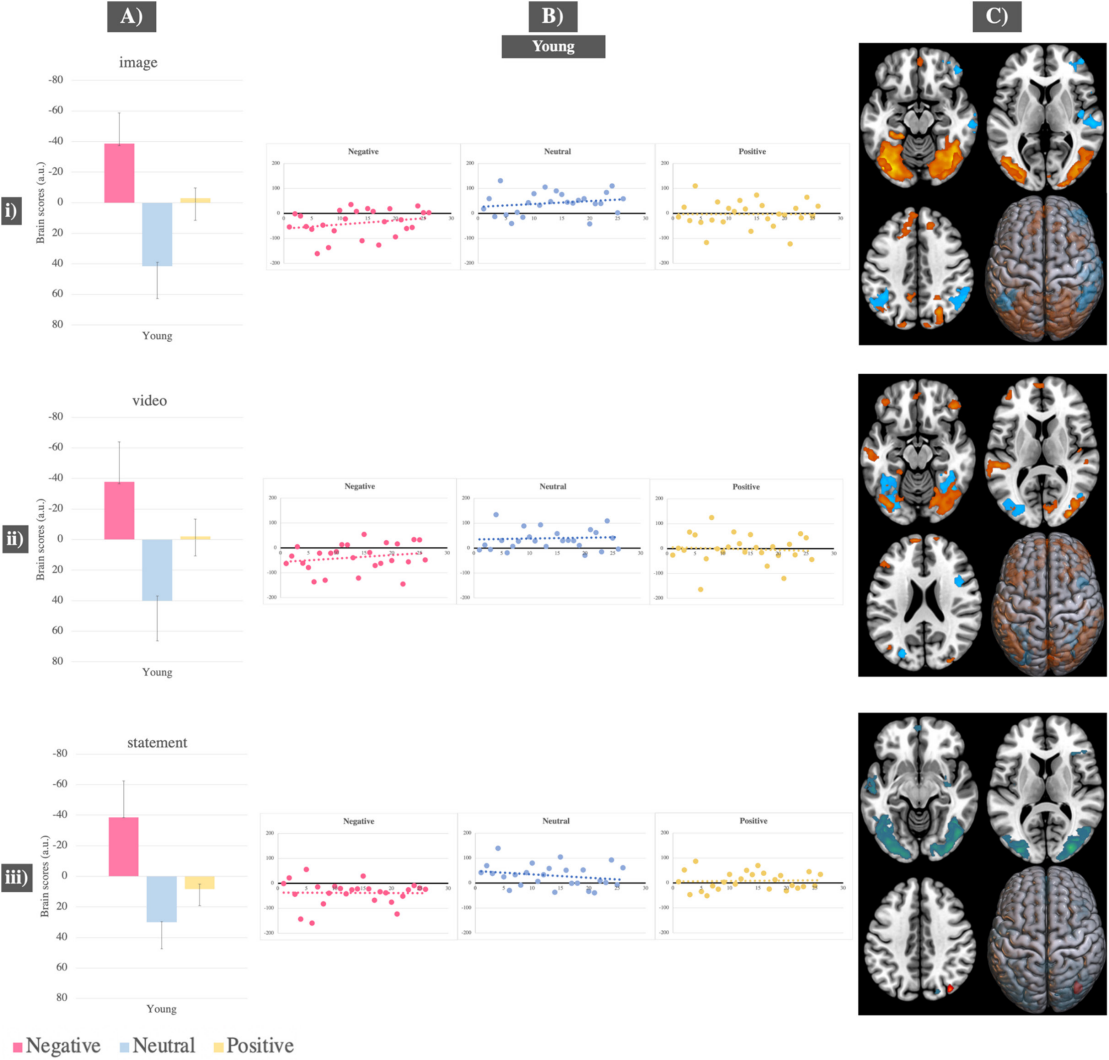
Task PLS results—Significant LVs in young adults only for: (i) image presentation; (ii) video exploration; (iii) episodic memory retrieval. (A) Mean brain scores related to activity in young adults for the negative versus neutral condition with error bars indicating 95% confidence intervals calculated from bootstrapping; (B) scatterplots displaying individual‐level brain scores for each condition and task stage, in young adults, highlighting differential neural patterns between the negative and neutral condition; (C) brain figures displaying regions with significantly increased activity in young adults for the negative condition (represented in red) and regions with significantly increased activity for the neutral condition (represented in blue).

In young adults, distinct brain activity patterns were identified during image and video presentation stages. One pattern was associated with the negative condition and another with the neutral condition during image presentation (*p* = 0.008, accounting for 76% covariance in the data) and video presentation (*p* = 0.026, accounting for 77% covariance in the data). The negative condition, compared to neutral, was associated with significantly greater activity in the inferior and middle occipital gyri, whereas the neutral condition, compared to negative, was associated with heightened activity in the fusiform and temporal gyri (see Figure 5i–ii and Tables S7–S8). Brain activity during the positive condition was not significantly different from either the neutral or negative conditions across these two stages, as indicated by the confidence intervals crossing 0. During memory retrieval, activity in the negative condition was differentiated from activity in the neutral and positive conditions (*p* = 0.02, accounting for 68% covariance in the data). The neutral and positive conditions, compared to the negative condition, were associated with activity in the right hippocampus, fusiform gyrus and areas in the inferior occipital gyri. The neutral condition activated these areas significantly more compared to the positive condition (see Figure 5iii and Table S9).

**FIGURE 6:**
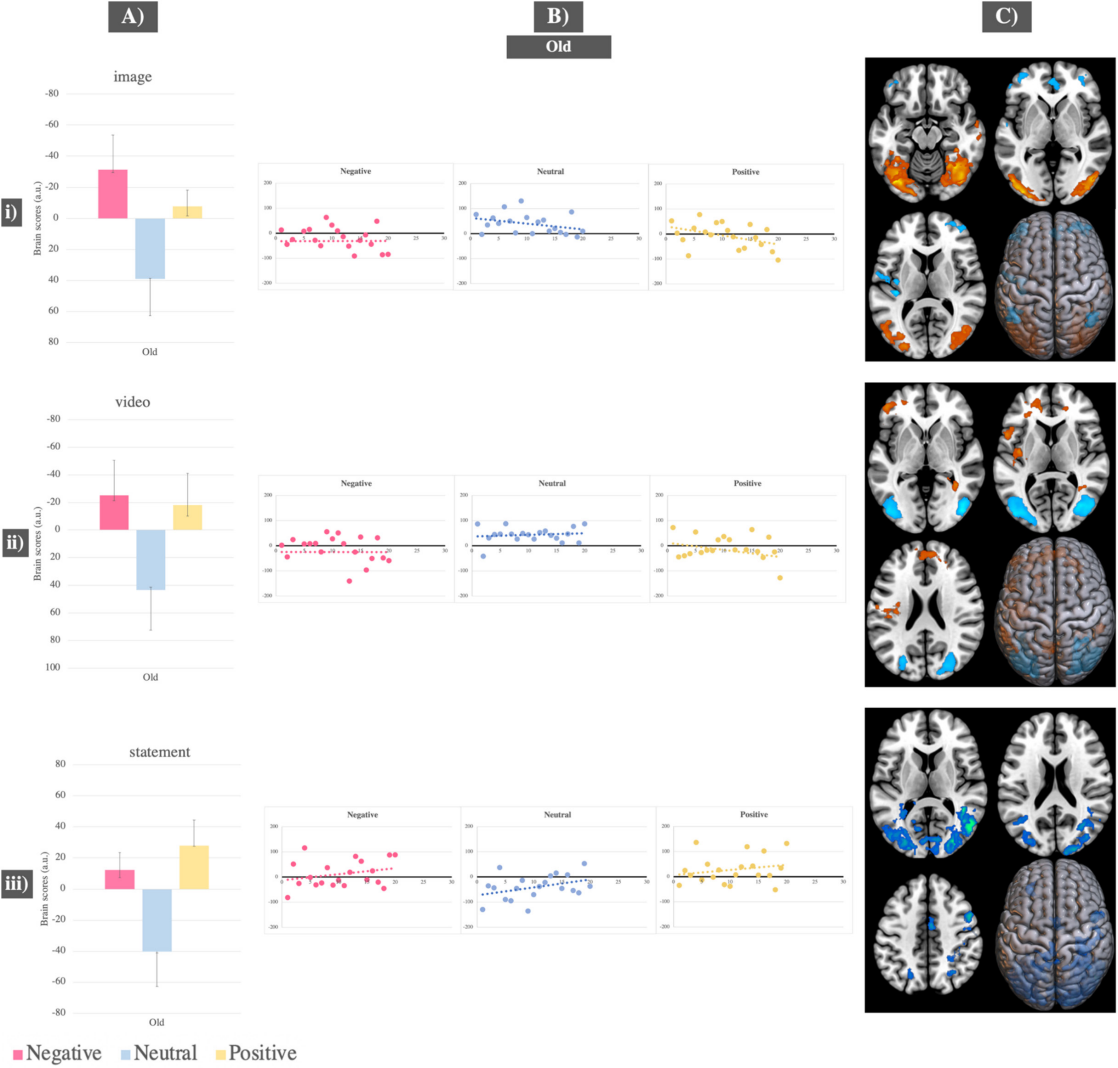
Task PLS results—Significant LVs in old adults only for: (i) image presentation; (ii) video exploration; (iii) episodic memory retrieval. (A) Mean brain scores related to activity in old adults for the positive and negative conditions compared to the neutral condition with error bars indicating 95% confidence intervals calculated from bootstrapping; (B) scatterplots displaying individual‐level brain scores for each condition and task stage in old adults, showing differential neural patterns between the valence conditions (negative and positive) and the neutral condition; (C) brain figures displaying regions with significantly increased activity in old adults for the high‐arousing conditions (negative and positive; represented in red) and regions with increased activity for the neutral condition (represented in blue).

In old adults, during image presentation (*p* = 0.006, accounting for 70% covariance in the data), video presentation (*p* = 0.024, accounting for 70% covariance in the data) and memory retrieval (*p* = 0.006, accounting for 62% covariance in the data), distinct neural patterns were found, one for the valence conditions (negative and positive) and one for the neutral condition. Activity changes were noted between the negative and positive conditions. During image presentation, the negative condition, compared to the positive condition, was associated significantly more with activity in the fusiform gyri, right temporal gyrus and left inferior occipital gyrus (see Figure 6i and Table S10). During video presentation, negative and positive conditions contributed equally and were associated with activity in the left superior medial frontal gyrus, left precuneus and inferior parietal gyrus (see Figure 6ii and Table S11). During retrieval, both positive and negative conditions were associated with decreased activity in the occipital and temporal gyri, compared to the neutral condition, with this decrease being more pronounced for the positive condition. The strength of neural activity prompted by the neutral condition did not change across the three task stages (see Figure 6iii and Table S12).

#### 3.2.2 Behavioural PLS Analyses

**FIGURE 7:**
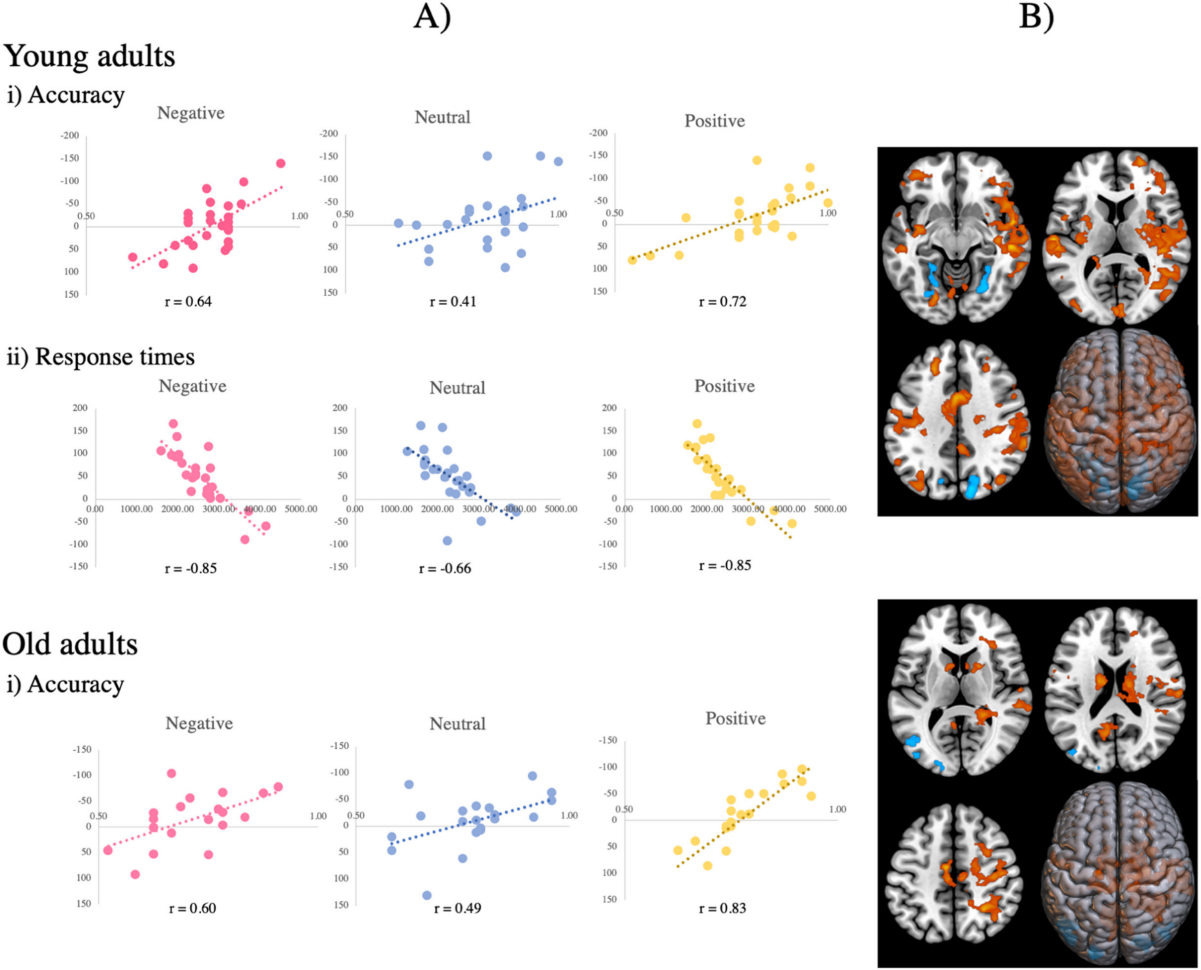
Behavioural PLS results showing correlations between whole‐brain activity and behavioural performance in: young and old adults separately across the three conditions. (A) Scatterplots show the relationship between averaged individual brain scores with (i) accuracy in young and old; and (ii) response times in only young. The correlation coefficient, denoted by the symbol ‘r’, quantifies the strength of these relationships. (B) Regions corresponding to areas activated more during better task performance, as indicated by better accuracy and faster response times, are highlighted in red, while regions corresponding to areas activated more during worse performance, as indicated by worse accuracy and slower response times, are highlighted in blue.

In young adults, the first behavioural PLS, relating accuracy to whole‐brain activity, revealed one significant LV (*p* = 0.03, accounting for 56% covariance in the data). In all three conditions, young adults who performed better on the task activated a right‐lateralised network, including the supramarginal gyrus, frontal, temporal and occipital gyri, in comparison to young adults who performed worse. The second behavioural PLS, relating response times to whole‐brain activity in young adults, showed one significant LV (*p* = 0.002, accounting for 69% covariance in the data). In all three conditions, faster performing young adults, compared to slower performing young adults, activated more the superior and middle temporal gyri, the right postcentral gyrus and left supplementary motor area (Figure 7 and Tables S13–S14).

In old adults, we found a brain pattern significantly relating to accuracy across all conditions (*p* = 0.05, accounting for 53% covariance in the data). Specifically, old adults, who performed better on the task, activated the supplementary motor area, post‐ and paracentral gyri significantly more than those who performed worse. Conversely, old adults, who performed worse on the task, showed increased activity in the occipital and middle temporal gyri (Figure 7 and Table S15). The final behavioural PLS relating response times to whole‐brain activity in old adults did not yield any significant LVs.

## 4 Discussion

This study aimed to investigate how emotional stimuli impact episodic memory for an adjacent valence‐free stimulus in young and old adults, on a behavioural and neural level. To achieve this, we developed a task that enabled the isolation of neural activity during exposure to emotional images, encoding of unrelated episodic memories through videos and retrieval of those episodic details. At the behavioural level, the study revealed that negative stimuli prompted worse performance during retrieval. At the neural level, findings showed valence‐related differences throughout the task, which were also driven by age. Exposure to positive valence was associated with a dynamic neural adaptation, unique to the old adult group.

The behavioural results showed that brief exposure to negative valence was associated with decreased accuracy and slower performance when retrieving information presented after the emotional stimulus, revealing a lingering effect of negative emotional valence. Previous studies indicate that negative stimuli capture and narrow attention (Astudillo et al. 2018; Bradley et al. 2011) and that the cognitive load accompanying negative information impacts related memories (e.g., Dolcos et al. 2017; Kensinger et al. 2007). Consistent with this, the current findings reveal that the cognitive load accompanying negative events extends to impact the retrieval of unrelated information. This highlights that even incidental negative emotions can disrupt memory processes, underscoring the importance of managing emotional influences on cognitive function. Regarding behavioural age‐related differences, accuracy performance was worse in old adults, aligning with established findings of age‐dependent decline in episodic memory (Kinugawa et al. 2013; Korkki et al. 2020). Notably, while previous studies have focused on rapid response tasks (e.g., Sharot et al. 2004), the current task used a generous 7 s response window for encoding and retrieval, which revealed no age‐related differences in response times. This suggests that when old adults are given sufficient time, they can perform comparably to young adults in terms of response speed. While old adults may typically exhibit smaller and more incremental adjustments in evidence accumulation (Wieschen et al. 2023), our task, which featured dynamic stimuli, contrasts with prior studies that used static stimuli (Sakaki et al. 2014; Sharot and Phelps 2004; St‐Laurent et al. 2011). This underscores that, under suitable conditions, old adults can maintain response time performance comparable to that of young adults.

The fMRI findings replicate age‐related differences in memory systems (St‐Laurent et al. 2011; Weeks et al. 2020). Specifically, the increased activity found in old adults, in regions associated with memory encoding, including the hippocampus, bilaterally, which plays a critical role in binding memory contexts (Yonelinas and Ritchey 2015), and the supplementary motor area, associated with planning and coordination (Nachev et al. 2008), suggests their increased engagement to compensate for declines in retrieval performance (Bangen et al. 2012). The increased hippocampal reliance may be a strategy to improve memory formation despite accuracy challenges (but see Stiernman et al. 2023). In contrast, the activity patterns observed in young adults in areas critical for memory processing, such as the middle temporal gyrus, and fusiform gyrus (Geiger et al. 2016; Ghuman et al. 2014) suggest more efficient retrieval of episodic information. Findings from the initial analysis showed that old adults were associated with increased neural activity during the encoding stages, compared to young adults who showed increased activity during the retrieval stage. This evidence aligns with the Compensation‐Related Utilisation of Neural Circuits Hypothesis (CRUNCH; Reuter‐Lorenz and Cappell 2008), as it reflects a compensatory increase in neural responses during lower task demands (i.e., while participants were just viewing images and videos), which broke down when task demands increased (i.e., during retrieval). The sudden decrease in neural activity during retrieval, in old compared to young adults, demonstrates the exact moment of ‘crunch’, which further justifies the worse accuracy performance observed in the older adult group. This study provides evidence to highlight a dynamic neural shift observed in ageing, across the course of a single task, under different levels of cognitive demands.

The neural evidence further revealed two key findings: (i) the sustained impact of negative valence on processes beyond encoding, as indicated by valence‐dependent differentiations across the task; and (ii) the unique influence of positive valence driven by age. Firstly, negative valence not only affected neural responses during exposure but also prolonged its effect to influence subsequent information encoding and retrieval. This finding is important as it helps justify the behavioural decline following negative valence by highlighting that the negative content of an experience can extend its effects, leaving a neural imprint influencing upcoming cognitive processes. The increased neural activity across both young and old adults, associated with negative compared to neutral valence, especially during video presentation, demonstrates a heightened neural effort to support encoding processes. These outcomes stress the necessity of assessing emotional factors during memory examinations. This is applicable for conditions such as post‐traumatic stress disorder, as emotional disturbances prompt memory disruptions (Isaac et al. 2022). The sustained effect of negative valence was not confined to young, as it persisted in old adults. The findings are in line with studies reporting heightened whole‐brain activity during retrieval in response to emotional stimuli (Dolcos et al. 2017). Moreover, they expand the understanding provided by the eCMR model (Talmi et al. 2019), showing that while increased arousal prompts emotional information binding with its source context, negative valence further leads to interference when processing subsequent unrelated information. In the present study, increased activity reflected a greater neural effort to support performance. This was further reinforced by the brain‐behaviour findings, which revealed that individuals with better, compared to those with worse memory performance, showed increased neural activity across a broad network. This network included the frontal gyri, associated with a strategic processing and decision‐making (Khader et al. 2011), middle temporal gyri and occipital cortices, reinforcing sensory engagement in visual memory retrieval (Vaidya et al. 2002). Together, these findings demonstrate the ongoing neural effects of negative valence, present in both young and old adults and further support the brain's increased effort to counteract negative‐valence–related effects, by increasing its activity.

Secondly, the current findings revealed neural differences in relation to positive valence, which were driven by age. Specifically, the young group exhibited unique neural responses between positive and negative valences during retrieval, whereas old adults did not exhibit this differentiation. This suggests an age‐related decline in the ability to selectively respond to emotional stimuli based on valence on a neural level (Martins et al. 2014). Findings are in line with the dedifferentiation theory in healthy ageing (Koen and Rugg 2019), implying a loss of neural specificity. The old adult brain therefore becomes less adept at assigning specific neural recruitment, leading to more generalised neural responses (Grady 2008). This neural generalisation was evident in old adults throughout the whole task. Interestingly, in the older group, even though both positive and negative valences activated the same regions, this neural activity was dynamically adjusted and driven by valence. Specifically, greater activity was evident in response to negative, compared to positive valence, immediately at the time of exposure. Over time, this increased activity for negative valence decreased, leading to equal activity levels between negative and positive valences during encoding. By the retrieval stage, positive valence contributed more to the reduction in neural activity compared to negative valence. This shift suggests that old adults initially responded more strongly to negative stimuli, but over time, positive stimuli had a stronger influence, indicating a compensatory mechanism reinforced by positive valence exposure. These findings align with research on emotion‐related differences in ageing (Sims et al. 2015; Urry and Gross 2010), which suggest that old adults tend to prefer positive over negative information (Carstensen and DeLiema 2018; Mammarella et al. 2016). The current study highlights the impact that even brief exposure to emotional stimuli has on unrelated upcoming memory processes. In the context of ageing, where shifts in emotional processing occur, these findings provide insights into how emotional experiences may modulate encoding and retrieval, suggesting a potential pathway for addressing age‐related memory decline.

One notable limitation of this study is the repetition of the videos throughout the task. Despite efforts to control and randomise the order of runs across participants, the design did not fully account for potential repetition effects. However, video repetition was included as a fixed factor in the mixed modelling analyses. This facilitated the systematic control for any confounding influences arising from exposure to the same stimuli. We acknowledge the relatively modest sample size, which may have potentially limited the ability to detect significant effects related to positive valence in some of our analyses. However, we believe that results would remain consistent with a larger sample size. First, by converting the behavioural data into z‐scores, we identified age‐related differences, demonstrating the reliability of our findings. Second, in the PLS analyses, bootstrapping was used to enhance the robustness of our estimates by utilising sample variance and leveraging assumptions of normality (Grady et al. 2015), thereby adding precision to our results. Finally, although no formal power analysis was conducted, the sample size was influenced by the statistical advantages of mixed‐effect models, which benefit from multiple observations per participant, enhancing the robustness of the analyses by increasing the total number of data points. Future studies could benefit from combining eye‐tracking with fMRI to gain deeper insights into how participants respond to negative stimuli. Analysing eye movements could help reveal whether negative valence influences video exploration, potentially leading to poorer memory retrieval.

Overall, episodic memory retrieval following valence exposure involves a synchronised effort across brain regions, in both young and old adults. The current study revealed that negative valence persists to affect memory processes behaviourally, and neurally, with negative events leading to worse retrieval in both groups. Although old adults generally showed a reduced ability to neurally distinguish between emotional valences, positive valence had a distinct effect in this group, with increased contribution from initial exposure through to retrieval, reflecting age‐related shifts in emotional processing. Overall, these findings demonstrate that even brief exposure to emotional valence can influence upcoming and unrelated cognitive processes, highlighting the interconnectedness of emotional processing and memory.

## Author Contributions

**Marianna Constantinou:** conceptualisation; data curation; formal analysis; investigation; methodology; visualisation; writing – original draft. **Ala Yankouskaya:** formal analysis; supervision; validation; writing – review and editing. **Hana Burianová:** methodology; project administration; supervision; validation; writing – review and editing. All authors have read and agreed to the current version of the manuscript.

## Ethics Statement

The study adhered to the ethical guidelines of Bournemouth University and received approval from the Bournemouth University Ethics Committee (protocol code 38758/40318).

## Conflicts of Interest

The authors declare no conflicts of interest.

### Peer Review

The peer review history for this article is available at https://www.webofscience.com/api/gateway/wos/peer‐review/10.1111/ejn.70041.

## Supporting information


**Table S1** Regions activated more in old compared to young adults, and regions activated more in young compared to old adults, during image presentation across all three conditions.
**Table S2.** Regions activated more in old compared to young adults, during video exploration across all three conditions.
**Table S3.** A region activated more in old compared to young adults, and regions activated more in young compared to old adults, during memory retrieval across all three conditions.
**Table S4.** Regions activated more during the presentation of a negative compared to a neutral image, in young and old adults, and regions activated more during the presentation of a neutral image, compared to a negative image in young and old adults.
**Table S5.** Regions activated more during video exploration in the negative (in young adults only) compared to the neutral condition (in young and old adults), and regions activated more for the neutral condition (in young and old adults), compared to negative condition (in young adults only).
**Table S6.** A region activated more during memory retrieval in the negative (in young adults only) compared to the neutral condition (in young and old adults), and regions activated more for the neutral condition (in young and old adults), compared to the negative condition (in young adults only).
**Table S7.** Regions activated more during the presentation of a negative image, compared to the neutral image, and regions activated more during the presentation of a neutral image compared to the presentation of a negative image, in young adults only.
**Table S8.** Regions activated more during video exploration for the negative condition, compared to the neutral condition, and regions activated more for the neutral condition compared to the negative condition, in young adults only.
**Table S9.** A region activated more during memory retrieval for the negative condition, compared to the positive and neutral conditions, and regions activated more for the positive and neutral conditions compared to the negative condition, in young adults only.
**Table S10.** Regions activated more during the presentation of negative and positive images, compared to the neutral image, and regions activated more during the presentation of a neutral image compared to the presentation of negative and positive images, in old adults only.
**Table S11.** Regions activated more during video exploration for the negative and positive conditions, compared to the neutral condition, and regions activated more for the neutral condition compared to the negative and positive conditions, in old adults only**.**
**Table S12.** Regions activated more during memory retrieval for the neutral condition compared to the negative and positive conditions, in old adults only.
**Table S13.** Regions significantly activated in better compared to worse performing young adults, across all conditions.
**Table S14.** Regions significantly activated in faster compared to slower performing young adults, and regions associated with slower compared to faster performing young adults across all conditions.
**Table S15.** Regions significantly activated in better compared to worse performing old adults, and regions associated with worse compared to better performing old adults across all conditions.

## Data Availability

Upon reasonable request, anonymised raw behavioural and imaging data will be made available.
