# Supplementary material for: Valence Effects on Episodic Memory in Young and Old Adults Following Exposure to Emotional Stimuli
Source: Eur J Neurosci. 2025 Mar 3;61(5):e70041. doi: 10.1111/ejn.70041 (PMC11875107; doi:10.1111/ejn.70041)
Supplement: Supplementary file 1 — Table S1 Regions activated more in old compared to young adults, and regions activated more in young compared to old adults, during image presentation across all three conditions. Table S2. Regions activated more in old compared to young adults, during video exploration across all three conditions. Table S3. A region activated more in old compared to young adults, and regions activated more in young compared to old adults, during memory retrieval across all three conditions. Table S4. Regions activated more during the presentation of a negative compared to a neutral image, in young and old adults, and regions activated more during the presentation of a neutral image, compared to a negative image in young and old adults. Table S5. Regions activated more during video exploration in the negative (in young adults only) compared to the neutral condition (in young and old adults), and regions activated more for the neutral condition (in young and old adults), compared to negative condition (in young adults only). Table S6. A region activated more during memory retrieval in the negative (in young adults only) compared to the neutral condition (in young and old adults), and regions activated more for the neutral condition (in young and old adults), compared to the negative condition (in young adults only). Table S7. Regions activated more during the presentation of a negative image, compared to the neutral image, and regions activated more during the presentation of a neutral image compared to the presentation of a negative image, in young adults only. Table S8. Regions activated more during video exploration for the negative condition, compared to the neutral condition, and regions activated more for the neutral condition compared to the negative condition, in young adults only. Table S9. A region activated more during memory retrieval for the negative condition, compared to the positive and neutral conditions, and regions activated more for the positive and neutral condit [file EJN-61-0-s001.docx]

| **Table 1.** Regions activated more in old compared to young adults, and regions activated more in young compared to old adults, during image presentation across all three conditions. | | | | | | | |
| --- | --- | --- | --- | --- | --- | --- | --- |
|  | H | Peak region | MNI coordinates | | | Voxel No. | BSR |
| Old > Young adults |  |  | x | y | z |  |  |
|  | R | Supplementary Motor Area | 2 | 0 | 50 | 1252 | 7.93 |
|  | R | Putamen | 32 | -12 | 0 | 7274 | 7.67 |
|  | L | Caudate Nucleus | -18 | -16 | 22 | 788 | 6.35 |
|  | R | Middle Frontal Gyrus | 48 | 46 | 8 | 762 | 5.96 |
|  | R | Cuneus | 12 | -76 | 32 | 1845 | 5.60 |
|  | R | Supramarginal Gyrus | 52 | -36 | 42 | 1437 | 5.29 |
| Young > Old adults |  |  |  |  |  |  |  |
|  | L | Middle Temporal Gyrus | -52 | 2 | -16 | 230 | -5.19 |
|  | R | Fusiform Gyrus | 28 | -56 | -8 | 514 | -5.07 |
|  | L | Middle Orbital Frontal Gyrus | -10 | 38 | -8 | 286 | -5.03 |

| **Table 2.** Regions activated more in old compared to young adults, during video exploration across all three conditions. | | | | | | | |
| --- | --- | --- | --- | --- | --- | --- | --- |
|  | H | Peak region | MNI coordinates | | | Voxel No. | BSR |
| Old > Young adults |  |  | x | y | z |  |  |
|  | L | Caudate Nucleus | -18 | -4 | 28 | 1059 | 6.27 |
|  | R | Hippocampus | 28 | -8 | -18 | 5420 | 6.13 |
|  | R | Inferior Temporal Gyrus | 60 | -56 | -14 | 106 | 5.82 |
|  | R | Lingual Gyrus | 2 | -70 | -2 | 149 | 5.28 |
|  | R | Middle Temporal Gyrus | 50 | -34 | -12 | 140 | 5.10 |
|  | L | Hippocampus | -36 | -40 | -4 | 144 | 4.83 |
|  |  | Cuneus | 0 | -84 | 26 | 308 | 4.81 |

| **Table 3.** A region activated more in old compared to young adults, and regions activated more in young compared to old adults, during memory retrieval across all three conditions. | | | | | | | |
| --- | --- | --- | --- | --- | --- | --- | --- |
|  | H | Peak region | MNI coordinates | | | Voxel No. | BSR |
| Old > Young adults |  |  | x | y | z |  |  |
|  | L | Middle Occipital Gyrus | -30 | -82 | 38 | 103 | 4.21 |
| Young > Old adults |  |  |  |  |  |  |  |
|  | L | Caudate Nucleus | -18 | -4 | 22 | 7372 | -7.10 |
|  | R | Supplementary Motor Area | 4 | 0 | 50 | 446 | -6.31 |
|  | R | Lingual Gyrus | 6 | -76 | -8 | 896 | -6.15 |
|  | R | Cuneus | 2 | -78 | 34 | 3087 | -5.97 |
|  | R | Precentral Gyrus | 50 | -10 | 50 | 921 | -5.86 |
|  | L | Lingual Gyrus | -4 | -66 | 0 | 1987 | -5.80 |
|  | L | Posterior Cingulum | -8 | -50 | 22 | 1941 | -5.74 |
|  | R | Fusiform Gyrus | 36 | -50 | -18 | 138 | -5.48 |
|  | R | Superior Temporal Gyrus | 54 | -10 | 6 | 2002 | -5.45 |

| **Table 4.** Regions activated more during the presentation of a negative compared to a neutral image, in young and old adults, and regions activated more during the presentation of a neutral image, compared to a negative image in young and old adults. | | | | | | | |
| --- | --- | --- | --- | --- | --- | --- | --- |
|  | H | Peak region | MNI coordinates | | | Voxel No. | BSR |
| Negative > Neutral |  |  | **x** | **y** | **z** |  |  |
|  | L | Fusiform Gyrus | -38 | -76 | -16 | 15384 | 12.54 |
|  | L | Inferior Occipital Gyrus | -44 | -80 | -6 | 4721 | 11.88 |
|  | R | Inferior Occipital Gyrus | 44 | -74 | -14 | 4829 | 11.07 |
|  | R | Precentral Gyrus | 44 | 12 | 20 | 159 | 5.42 |
|  | L | Precentral Gyrus | -40 | 0 | 28 | 126 | 4.84 |
| Neutral > Negative |  |  |  |  |  |  |  |
|  | R | Supramarginal Gyrus | 62 | -46 | 36 | 1103 | -7.16 |
|  | L | Inferior Parietal Gyrus | -40 | -50 | 38 | 1299 | -6.51 |
|  | R | Superior Temporal Gyrus | 60 | -26 | 8 | 901 | -6.22 |

| **Table 5.** Regions activated more during video exploration in the negative (in young adults only) compared to the neutral condition (in young and old adults), and regions activated more for the neutral condition (in young and old adults), compared to negative condition (in young adults only). | | | | | | | |
| --- | --- | --- | --- | --- | --- | --- | --- |
|  | H | Peak region | MNI coordinates | | | Voxel No. | BSR |
| Negative young> Neutral both | |  | **x** | **y** | **z** |  |  |
|  | L | Precuneus | -10 | -74 | 58 | 128 | 6.67 |
|  | L | Insula | -38 | -18 | 16 | 201 | 5.93 |
|  | L | Middle Temporal Gyrus | -50 | -32 | 4 | 479 | 5.90 |
|  | R | Superior Occipital Gyrus | 36 | -76 | 46 | 302 | 5.80 |
| Neutral both > Negative young | |  |  |  |  |  |  |
|  | L | Inferior Occipital Gyrus | -40 | -74 | -10 | 3065 | -8.88 |
|  | R | Inferior Occipital Gyrus | 40 | -64 | -14 | 2871 | -8.26 |
|  | R | Fusiform Gyrus | 36 | -54 | -14 | 1655 | -7.46 |
|  | L | Middle Occipital Gyrus | -44 | -82 | 12 | 2213 | -7.05 |
| **Table 6.** A region activated more during memory retrieval in the negative (in young adults only) compared to the neutral condition (in young and old adults), and regions activated more for the neutral condition (in young and old adults), compared to the negative condition (in young adults only). | | | | | | | |
|  | H | Peak region | MNI coordinates | | | Voxel No. | BSR |
| Negative young > Neutral both | |  | **x** | **y** | **z** |  |  |
|  | R | Middle Occipital Gyrus | 38 | -76 | 36 | 127 | -5.11 |
| Neutral both> Negative young | |  |  |  |  |  |  |
|  | R | Inferior Occipital Gyrus | 44 | -74 | -12 | 3609 | 9.08 |
|  | R | Middle Occipital Gyrus | 46 | -78 | 4 | 2504 | 8.13 |
|  | L | Inferior Occipital Gyrus | -38 | -74 | -10 | 3459 | 7.88 |
|  | L | Middle Occipital Gyrus | -28 | -90 | 0 | 3325 | 7.00 |

| **Table 7.** Regions activated more during the presentation of a negative image, compared to the neutral image, and regions activated more during the presentation of a neutral image compared to the presentation of a negative image, in young adults only. | | | | | | | |
| --- | --- | --- | --- | --- | --- | --- | --- |
|  | H | Peak region | MNI coordinates | | | Voxel No. | BSR |
| Negative > Neutral |  |  | **x** | **y** | **z** |  |  |
|  | L | Lateral Occipital Gyrus | -44 | -80 | -14 | 3349 | -12.47 |
|  | R | Superior Parietal Gyrus | 28 | -56 | 50 | 651 | -11.21 |
|  | L | Inferior Occipital Gyrus | -44 | -78 | -14 | 5355 | -10.73 |
|  | R | Inferior Occipital Gyrus | 42 | -68 | -14 | 5501 | -9.79 |
| Neutral > Negative |  |  |  |  |  |  |  |
|  | R | Superior Temporal Gyrus | 56 | -26 | 10 | 439 | 6.60 |
|  | R | Middle Temporal Gyrus | 64 | -24 | -12 | 414 | 5.55 |
|  | R | Middle Frontal Gyrus | 42 | 52 | 4 | 307 | 5.21 |
|  | L | Inferior Parietal Gyrus | -40 | -50 | 40 | 533 | 5.05 |

| **Table 8.** Regions activated more during video exploration for the negative condition, compared to the neutral condition, and regions activated more for the neutral condition compared to the negative condition, in young adults only. | | | | | | | |
| --- | --- | --- | --- | --- | --- | --- | --- |
|  | H | Peak region | MNI coordinates | | | Voxel No. | BSR |
| Negative > Neutral |  |  | x | y | z |  |  |
|  | R | Angular Gyrus | 60 | -50 | 38 | 488 | -6.46 |
|  | L | Inferior Occipital Gyrus | -42 | -74 | -10 | 1329 | -6.44 |
|  | R | Middle Occipital Gyrus | 34 | -90 | 10 | 2326 | -6.18 |
|  | L | Superior Temporal Gyrus | -46 | -32 | 12 | 729 | -5.63 |
| Neutral > Negative |  |  |  |  |  |  |  |
|  | L | Fusiform Gyrus | -34 | -76 | -12 | 1644 | 6.86 |
|  | R | Inferior Occipital Gyrus | 40 | -62 | -14 | 1102 | 6.04 |
|  | R | Inferior Operculum Frontal Gyrus | 44 | 10 | 26 | 155 | 5.88 |
|  | R | Fusiform Gyrus | 38 | -52 | -14 | 786 | 5.85 |

| **Table 9.** A region activated more during memory retrieval for the negative condition, compared to the positive and neutral conditions, and regions activated more for the positive and neutral conditions compared to the negative condition, in young adults only. | | | | | | | |
| --- | --- | --- | --- | --- | --- | --- | --- |
|  | H | Peak region | MNI coordinates | | | Voxel No. | BSR |
| Negative > Neutral & Positive | |  | **x** | **y** | **z** |  |  |
|  | R | Middle Occipital Gyrus | 38 | -78 | 38 | 128 | -5.62 |
| Neutral & Positive > Negative | |  |  |  |  |  |  |
|  | L | Inferior Occipital Gyrus | -36 | -70 | -4 | 1999 | 7.82 |
|  | R | Inferior Occipital Gyrus | 46 | -62 | -12 | 2220 | 7.58 |
|  | L | Superior Temporal Gyrus | -50 | -6 | -6 | 340 | 7.41 |
|  | R | Middle Occipital Gyrus | 34 | -84 | 16 | 1347 | 7.12 |
|  | R | Fusiform Gyrus | 44 | -60 | -16 | 1153 | 7.09 |
|  | R | Hippocampus | 34 | -14 | -14 | 200 | 6.17 |

| **Table 10.** Regions activated more during the presentation of negative and positive images, compared to the neutral image, and regions activated more during the presentation of a neutral image compared to the presentation of negative and positive images, in old adults only. | | | | | | | |
| --- | --- | --- | --- | --- | --- | --- | --- |
|  | H | Peak region | MNI coordinates | | | Voxel No. | BSR |
| Negative & Positive > Neutral | |  | **x** | **y** | **z** |  |  |
|  | R | Fusiform Gyrus | 34 | -58 | -16 | 2492 | -9.11 |
|  | L | Inferior Occipital Gyrus | -42 | -76 | -12 | 2362 | -8.80 |
|  | L | Fusiform Gyrus | -38 | -72 | -14 | 1931 | -8.67 |
|  | R | Middle Temporal Gyrus | 64 | -16 | -12 | 117 | -6.80 |
| Neutral > Negative & Positive | |  |  |  |  |  |  |
|  | L | Transverse Temporal Gyrus | -38 | -24 | 12 | 270 | 6.96 |
|  | R | Angular Gyrus | 46 | -64 | 48 | 283 | 5.64 |
|  | L | Angular Gyrus | -48 | -60 | 48 | 298 | 5.27 |
|  | L | Middle Frontal Gyrus | -36 | 54 | 2 | 257 | 5.24 |
|  | R | Anterior Cingulum | 6 | 44 | 2 | 117 | 5.08 |

| **Table 11.** Regions activated more during video exploration for the negative and positive conditions, compared to the neutral condition, and regions activated more for the neutral condition compared to the negative and positive conditions, in old adults only**.** | | | | | | | |
| --- | --- | --- | --- | --- | --- | --- | --- |
|  | H | Peak region | MNI coordinates | | | Voxel No. | BSR |
| Negative & Positive > Neutral | |  | **x** | **y** | **z** |  |  |
|  | L | Superior Medial Frontal Gyrus | -14 | 42 | 6 | 131 | -7.53 |
|  | L | Precuneus | -8 | -56 | 42 | 205 | -6.13 |
|  | L | Inferior Parietal Gyrus | -54 | -54 | 48 | 154 | -5.53 |
| Neutral > Negative & Positive | |  |  |  |  |  |  |
|  | R | Middle Occipital Gyrus | 42 | -76 | 4 | 2427 | 8.26 |
|  | L | Middle Occipital Gyrus | -44 | -78 | 2 | 2350 | 7.94 |
|  | R | Middle Temporal Gyrus | 46 | -70 | 16 | 897 | 5.38 |
|  | R | Superior Parietal Gyrus | 36 | -52 | 58 | 126 | 4.48 |

| **Table 12.** Regions activated more during memory retrieval for the neutral condition compared to the negative and positive conditions, in old adults only. | | | | | | | |
| --- | --- | --- | --- | --- | --- | --- | --- |
|  | H | Peak region | MNI coordinates | | | Voxel No. | BSR |
| Neutral > Negative & Positive | |  | **x** | **y** | **z** |  |  |
|  | R | Middle Temporal Gyrus | 48 | -68 | 8 | 4253 | -9.43 |
|  | R | Inferior Temporal Gyrus | 48 | -70 | -6 | 2464 | -8.97 |
|  | R | Inferior Occipital Gyrus | 44 | -80 | -2 | 1829 | -8.28 |
|  | L | Inferior Occipital Gyrus | -42 | -70 | -4 | 2108 | -8.15 |
|  | L | Fusiform Gyrus | -42 | -72 | -14 | 2320 | -7.65 |

| **Table 13.** Regions significantly activated in better compared to worse performing young adults, across all conditions. | | | | | | | |
| --- | --- | --- | --- | --- | --- | --- | --- |
|  | H | Peak region | MNI coordinates | | | Voxel No. | BSR |
| Better > Worse accuracy | |  | **x** | **y** | **z** |  |  |
|  | L | Inferior Operculum Frontal Gyrus | -42 | 14 | 22 | 161 | -8.51 |
|  | L | Middle Occipital Gyrus | -46 | -72 | 18 | 766 | -8.17 |
|  | R | Superior Frontal Gyrus | 24 | 60 | 8 | 104 | -7.20 |
|  | R | Middle Temporal Gyrus | 50 | -68 | 18 | 648 | -6.91 |
|  | R | Middle Occipital Gyrus | 42 | -72 | 32 | 127 | -6.62 |
|  | R | Middle Frontal Gyrus | 30 | 28 | 52 | 198 | -6.42 |
|  | R | Supramarginal Gyrus | 64 | -40 | 32 | 374 | -6.36 |
|  | R | Postcentral Gyrus | 14 | -40 | 78 | 350 | -6.24 |
|  | R | Inferior Orbital Frontal Gyrus | 50 | 36 | -4 | 259 | -6.23 |

| **Table 14.** Regions significantly activated in faster compared to slower performing young adults, and regions associated with slower compared to faster performing young adults across all conditions. | | | | | | | |
| --- | --- | --- | --- | --- | --- | --- | --- |
|  | H | Peak region | MNI coordinates | | | Voxel No. | BSR |
| Faster > Slower performance | |  | **x** | **y** | **z** |  |  |
|  | R | Postcentral Gyrus | 50 | -24 | 46 | 1121 | 11.27 |
|  | R | Superior Temporal Gyrus | 52 | 0 | 0 | 7824 | 8.91 |
|  | L | Superior Temporal Gyrus | -62 | -20 | 12 | 974 | 8.90 |
|  | L | Supplementary Motor Area | -4 | 2 | 48 | 699 | 7.90 |
|  | R | Middle Temporal Gyrus | 52 | -26 | -10 | 562 | 7.88 |
| Slower > Faster performance | |  |  |  |  |  |  |
|  | R | Cuneus | 14 | -82 | 36 | 383 | -7.44 |
|  | R | Fusiform Gyrus | 26 | -64 | -12 | 236 | -6.25 |
|  | L | Fusiform Gyrus | -22 | -52 | -14 | 160 | -6.18 |
|  | L | Superior Occipital Gyrus | -16 | -74 | 30 | 139 | -5.73 |

| **Table 15.** Regions significantly activated in better compared to worse performing old adults, and regions associated with worse compared to better performing old adults across all conditions. | | | | | | | |
| --- | --- | --- | --- | --- | --- | --- | --- |
|  | H | Peak region | MNI coordinates | | | Voxel No. | BSR |
| Better > Worse performance | |  | **x** | **y** | **z** |  |  |
|  | L | Supplementary Motor Area | -6 | -10 | 50 | 1265 | -8.86 |
|  | R | Cingulate Gyrus | 14 | -32 | 40 | 113 | -7.42 |
|  | L | Postcentral Gyrus | -52 | -8 | 26 | 171 | -7.33 |
|  | L | Paracentral Gyrus | -18 | -28 | 70 | 287 | -7.27 |
| Worse > Better performance | |  |  |  |  |  |  |
|  | L | Middle Occipital Gyrus | -22 | -94 | 16 | 151 | 7.06 |
|  | L | Middle Temporal Gyrus | -52 | -70 | 4 | 359 | 6.85 |
|  | R | Inferior Occipital Gyrus | 44 | -76 | -2 | 151 | 5.66 |
|  | R | Lingual Gyrus | 18 | -82 | -4 | 174 | 5.52 |
| Abbreviations: BSR = bootstrap ratio (salience/SE ratio from the bootstrap analysis); H = hemisphere; L = left; R = right; Voxel No. = Number of Voxels; x coordinate = right/left; y coordinate = anterior/posterior; z coordinate = superior/inferior. | | | | | | | |
